# Is There a Relationship between the Stratum Corneum Thickness and That of the Viable Parts of Tumour Cells in Basal Cell Carcinoma?

**DOI:** 10.1155/2016/6146091

**Published:** 2016-01-28

**Authors:** Olav A. Foss, Patricia Mjønes, Silje Fismen, Eidi Christensen

**Affiliations:** ^1^Orthopaedic Research Centre, Clinic of Orthopaedy, Rheumatology and Dermatology, St. Olavs Hospital, Trondheim University Hospital, Trondheim 7030, Norway; ^2^Department of Cancer Research and Molecular Medicine, Faculty of Medicine, Norwegian University of Science and Technology (NTNU), Trondheim 7030, Norway; ^3^Department of Pathology and Medical Genetics, St. Olavs Hospital, Trondheim University Hospital, Trondheim 7030, Norway; ^4^Department of Pathology, University Hospital of North Norway, Tromsø 9019, Norway; ^5^Department of Dermatology, Clinic of Orthopaedy, Rheumatology and Dermatology, St. Olavs Hospital, Trondheim University Hospital, Trondheim 7030, Norway

## Abstract

Basal cell carcinoma (BCC) is an invasive epithelial skin tumour. The thickness of the outermost epidermal layer of the skin, the stratum corneum (SC), influences drug uptake and penetration into tumour and may thereby affect the response of BCC to topical treatment. The aim was to investigate a possible relationship between the thickness of the SC and that of the viable part of BCC. Histopathological evaluations of the corresponding SC and viable tumour thickness measurements of individual BCCs of different subtypes were explored. A total of 53 BCCs from 46 patients were studied. The median tumour thickness was 1.7 mm (0.8–3.0 mm), with a significant difference between subtypes (*p* < 0.001). The SC had a median thickness of 0.3 mm (0.2–0.4 mm), with no difference between tumour subtypes (*p* = 0.415). Additionally, no significant association between the thickness of the SC and that of the viable part of the tumour was demonstrated (*p* = 0.381). In conclusion our results indicate that SC thickness is relatively constant in BCC.

## 1. Introduction

The stratum corneum (SC) is the outermost epidermal layer of the skin. It consists of flattened, anucleated keratinized cells (corneocytes) enclosed in lipid bilayers including ceramides, free fatty acids, and cholesterol which together with enzymes, antimicrobial peptides, and structural proteins make a barrier function [1].

Basal cell carcinoma (BCC) is an epithelium tumour that primarily originates in the epidermis and its appendages. It is the most common type of invasive skin cancer in the fair skinned population of the world, causing significant patient morbidity, and should be managed properly [2]. Several types of treatment can be used of which minimally invasive methods such as topical photodynamic therapy (PDT) have become an attractive option [3]. This method is recommended for treatment of superficial BCCs and for small nodular tumours. However, the treatment response of thick tumours is regarded as inferior, partly because of the limited penetration of topical drugs through the SC, and deep into the tumour [4]. The SC physiochemical properties provide the main barrier for drug penetration of the skin [1]. In addition, crusts may cover part or the whole of the BCC, thereby increasing the thickness of the outer barrier layer.

SC thickness has been closely investigated in normal skin and has been found to vary, depending on various factors such as body site [5, 6]. However, the knowledge of SC thickness in BCC as well as information as to whether its thickness varies with total tumour thickness and across tumour subtypes is lacking.

A clinical estimate of BCC thickness can readily be made before selecting an appropriate therapy. Nevertheless, it is the histopathological thickness that is considered the “gold standard” measurement for prediction of treatment response. Information about the agreement between clinical and histopathological evaluations of BCC thickness is, to our knowledge, limited to the results of a single study [7]. In that study, the estimation of tumour thickness by the two methods was poor between corresponding measurements for individual tumours. It should be noted that the clinical estimation of tumour thickness includes the SC and any overlying crusts, as opposed to histology. In accordance with standard practice, the histological thickness is measured from the upper part of the stratum granulosum (SG) and comprises the viable part of the tumour cells [8]. Hence, there is a systematic difference between clinical and histopathological evaluations of BCC thickness, which may bias comparisons of results between these two methods. For this reason, and because the SC is the main barrier to percutaneous penetration of topically applied drugs, we wanted to investigate SC thickness in BCC.

The main objective of the present study was to investigate a possible relationship between measurements of SC thickness and the thickness of the corresponding viable part of the BCC.

## 2. Materials and Methods

The study was performed at the outpatient clinic at the Department of Dermatology, St. Olavs Hospital, Trondheim University Hospital (Trondheim, Norway), and approved by the regional committee for medical research ethics (REK number 4.2007.558). Patients gave written informed consent before entry into the study.

Part of the study sample had been included in previous reports that compared measurements of BCC thickness from clinical investigations, punch biopsies, and excision specimens [7, 9]. Consecutive patients of both sexes, over 18 years of age, with primary histopathologically verified BCC suitable for excision surgery and over 9 mm in size to ensure sufficient material for investigation, were included. Pregnancy and lactation were exclusion criteria.

Three physicians (two dermatology consultants and an experienced dermatology registrar) performed the clinical examinations of, respectively, 12, 17, and 24 tumours. A single hospital pathologist performed the histopathological assessment of all the specimens.

Clinical evaluations and sampling of tumour tissue for histopathological investigations were performed on the same day. The tumour size (in mm) was clinically defined as the mean value of its measured maximum length and width. Details of study inclusions and exclusions, the particular tissue sampling procedures, and the tissue processing are given in an earlier report [9].

BCC thickness was measured on haematoxylin, eosin, and saffron- (HES-) stained slides using an ocular micrometer (Vernier method) with a precision of 0.1 mm [10]. The BCC-free deep margin was defined as at least 0.1 mm of tumour-free tissue. The thicknesses were measured with the upper part of the SG as reference. The thickness of the viable part of the tumour was measured from this reference position to the bottom of the tumour nest and is referred to in this paper as the “tumour thickness.” The thickness of the SC with any crust was measured from the reference position to the outermost surface and is referred to as the “SC thickness.” The greatest thickness measurements for both the SC and the viable part of each tumour were used in the analysis as this was considered most relevant to the use of topical therapy. The tumours were histopathologically subclassified into three subtypes: superficial, nodular, and aggressive. The aggressive category included morpheaform, infiltrative, and basosquamous types [11]. They were classified according to the most aggressive component for those BCCs representing a mixed growth pattern.

All statistical calculations were performed using IBM SPSS Statistics (v.21, IBM Corp., Armonk, NY, USA). Visual inspection of Q-Q plots was used to examine whether data were normally distributed. The tumour size data were normally distributed and are presented as mean (SD). The tumour thickness measurements were normally distributed, whereas the SC thickness measurements were not. Therefore, all thickness data are presented as medians (quartiles). The Kruskal-Wallis test was used to compare SC thickness between subtypes. Regression analysis with tumour thickness and SC thickness was carried out to describe a possible relationship between these two parameters. A mixed linear model was used. All thickness measurement data had to be logarithmically transformed to achieve the assumption of normally distributed residuals in the models. Tumour size, location, and patient sex and age were initially included as covariates. For all tests, *p* < 0.05 was considered statistically significant.

## 3. Results

Measurements were taken of 53 BCCs from 46 patients. Of these tumours, 26 were located on the head/neck, 27 on the trunk, and six on the extremities. Histopathologically, 14 were superficial, 24 were nodular, and 15 were aggressive tumours. Twelve of the BCCs demonstrated crusts on histology.

**Figure 1:**
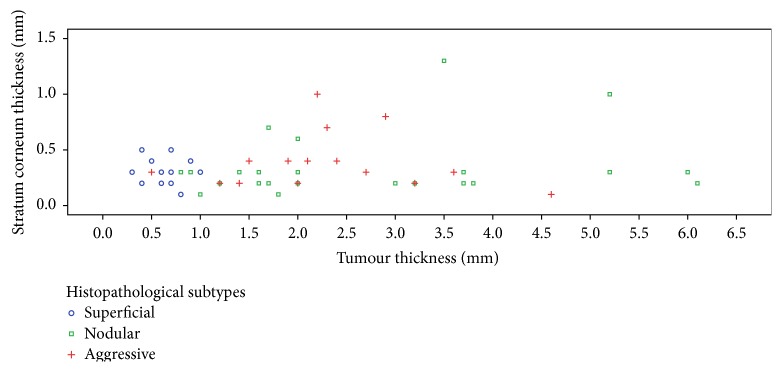
Plot of corresponding stratum corneum thickness versus tumour thickness of individual BCCs.

**Figure 2:**
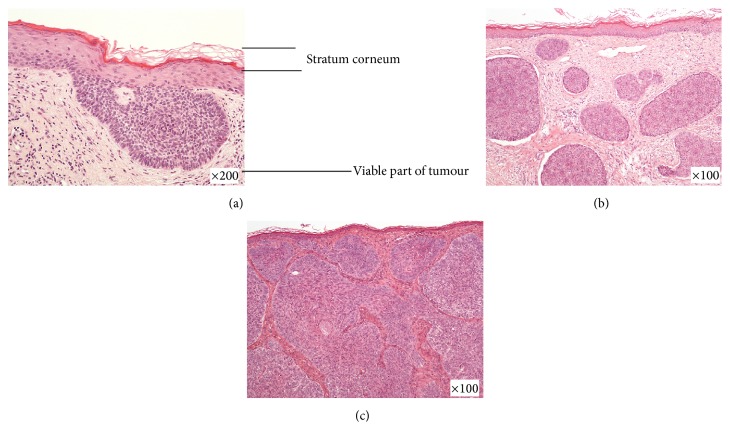
HES-stained histopathological images of sections of the stratum corneum and the viable parts of tumour cells from three basal cell carcinomas with different growth patterns: (a) superficial tumour from the back, (b) nodular tumour from the chest, and (c) aggressive tumour from the cheek.

The descriptive data are presented in Table 1. The median tumour thickness was 1.7 mm (0.8–3.0 mm) with a significant difference between subtypes (*p* < 0.001). Superficial tumours were significantly thinner than nodular and aggressive types (both *p* < 0.001), and there was no difference between the nodular and aggressive types (*p* = 0.813). The median SC thickness was 0.3 mm (0.2–0.4 mm) with no significant difference between subtypes (*p* = 0.415).  Figure 1 presents a scatter plot of tumour thickness and SC thickness. The regression analyses showed no statistically significant association between the tumour and SC thicknesses (*p* = 0.381). Three examples of BCCs of different subtype with different thicknesses for the viable cellular part but with similar SC thicknesses are shown in Figure 2. None of the following parameters were significant when included as covariates in the regression analyses: tumour size (*p* = 0.432), tumour location (*p* = 0.992), patient sex (*p* = 0.497), and patient age (*p* = 0.776).

## 4. Discussion

The main finding was the no significant relationship between the thickness of the SC and that of the viable part BCC, regardless of tumour size and subtype.

Knowledge of SC thickness in skin cancer is of interest in relation to PDT and may also be of interest to other topical therapies. PDT exerts its action by light activation of a photosensitizer, leading to destruction of targeted tumour cells. 5-Aminolaevulinic acid (ALA) and its ester derivate are the two most commonly used prodrugs for topical PDT of BCC [4]. After cellular uptake, ALA is metabolized to photosensitive porphyrins, and any factors limiting its penetration through skin may reduce the treatment effect. The thickness of SC has been shown to influence local uptake of ALA in viable cells [12, 13]. This was studied using fluorescence diagnosis with ALA-induced porphyrins in psoriatic plaque and actinic keratosis [14, 15]. When excited by blue light, porphyrin accumulating cells emit red fluorescence that is visualized. The SC proved thicker in lower fluorescent psoriatic plaques and a negative correlation between SC thickness and fluorescence intensity in actinic keratosis was observed. These findings indicate that SC thickness is a factor in differences of ALA uptake in viable cells and this may in turn be significant for treatment outcome. Thus, it was of interest to investigate whether the SC thickness varies in different subtypes of BCC when PDT efficacy is more superior in superficial than other subtypes of tumour. Although slight variation in SC thicknesses was observed between individual BCCs (lower quartile 0.2 mm, upper quartile 0.4 mm), the SC thickness does not appear to explain different treatment results on a group level between tumour subtypes.

In accordance with current PDT guidelines, SC and any crusts are commonly removed from the tumour surface and surrounding skin area before treatment in attempt to enhance drug permeation into tumour tissue [16, 17]. Various physical and/or chemical methods may be used [17, 18]. However, pre-PDT preparation can produce varying results because the procedures are, at present, not standardized and depend on both methods and physicians' assessments, aims, and skills.

We also wanted to examine whether SC thickness is a factor that may affect clinical assessment of tumour thickness. In an earlier aforementioned study, the results demonstrated that the clinicians overestimated the thickness of thin tumours and underestimated the thickness of thick tumours compared to histology [7]. The histopathological thickness was measured from the upper SG, whereas the clinical estimation of thickness included the SC and any crusts, as the clinician could not distinguish between microscopic tissue layers. The present results imply that the SC thickness probably could have contributed to the clinical overestimation of the thickness of thin BCCs.

The thickness of the SC in nonmelanoma skin cancers such as* in situ* and invasive squamous cell carcinomas has been studied previously, and no significant difference in SC thickness between the different histological diagnoses was shown [12]. Our results are in agreement, as we found no difference in SC thickness across BCC subtypes. However, in normal skin SC thickness may vary depending on body site, and within-site variation has also been observed [19]. Results from investigations of relationship between the SC thickness and sex and age in humans have been contradictory [5, 6, 20]. In the present study, these factors were initially included as covariates in the regression analysis, and none of them was found to be significant. However, our findings apply only to the particular analyses described in this report and should not be considered as contradicting the findings presented in the earlier cited reports. The relatively small number of tumours included should be regarded as a study limitation, particularly with regard to subgroup analyses. Other factors can influence the results. It is important for accurate measurement of thickness that the cuts from the tissue blocks were taken perpendicular to the skin surface. Also, possible errors in thickness estimations may have occurred owing to alterations in the physical properties of tissue after excision and preparation and individual evaluation of specimens are subject to variation [6, 21, 22].

## 5. Conclusions

The study results indicate that the thickness of SC in BCC is fairly constant independent of the viable tumour thickness and across different tumour sizes and subtypes.

## Figures and Tables

**Table 1:** Descriptive data of patients and BCCs.

Tumour type	Age [years]	Sex [M/F]	Number	Location [H/T/E]	Tumour size [mm] Mean (SD)	Tumour thickness [mm]	Stratum corneum thickness [mm]
						Median (quartiles)	
Superficial	69	7/7	14	2/9/3	17 ± 5	0.6 (0.4–0.7)	0.3 (0.2–0.4)
Nodular	76	17/7	24	12/10/2	18 ± 6	2.0 (1.6–3.7)	0.2 (0.2–0.3)
Aggressive	73	9/6	15	6/8/1	18 ± 4	2.2 (1.5–2.9)	0.3 (0.2–0.4)
All	73	33/20	53	20/27/6	18 ± 5	1.7 (0.8–3.0)	0.3 (0.2–0.4)

E: extremities; F: female; H: head/neck; M: male; SD: standard deviation; T: trunk.
